# Acute Intermittent Porphyria: A Diagnostic Conundrum

**DOI:** 10.7759/cureus.72419

**Published:** 2024-10-26

**Authors:** Vijay Balaji Vadivel, Simran Lamba

**Affiliations:** 1 Acute Medicine, Cumberland Infirmary, North Cumbria Integrated Care, NHS Trust, Carlisle, GBR; 2 Internal Medicine, ABLE Charitable Hospital, Bahrola, IND

**Keywords:** abdominal pain, acute intermittent porphyria, autosomal dominant disorder, porphobilinogen, siadh

## Abstract

This case report presents the case of a 20-year-old female patient who sought emergency medical attention for severe abdominal pain, nausea and vomiting, tachycardia, hypertension, and discolored urine. Initial diagnostic evaluations yielded no significant abnormalities; however, subsequent analysis revealed elevated urinary porphobilinogen, corroborating a diagnosis of acute intermittent porphyria (AIP). The patient’s medical history included recurrent urinary tract infections and a prior episode of syndrome of inappropriate antidiuretic hormone secretion (SIADH), in conjunction with psychiatric comorbidities of anxiety and depression.

Management encompassed a multifaceted approach involving supportive therapies, such as hydration and analgesia, alongside the imperative to abstain from using contraindicated pharmacological agents. Following referral to the National Acute Porphyria Service (NAPS), the patient received intravenous Haem arginate, resulting in clinical improvement and subsequent discharge. Nonetheless, she later necessitated further hospitalization due to the recurrence of similar symptoms.

This case highlights the exigency of recognizing AIP in young women presenting with nonspecific symptoms, necessitating a high index of clinical suspicion. Furthermore, it accentuates the critical importance of early specialist intervention to avert severe sequelae associated with acute episodes. The integration of targeted educational initiatives within Acute Medicine departments is paramount for fostering awareness and facilitating prompt diagnosis and management of this rare yet significant disorder.

## Introduction

Acute intermittent porphyria (AIP) usually presents with nonspecific symptoms, which often leads to a delay in diagnosis. The diagnosis of this case in a rural District General Hospital required a high index of suspicion in a young woman presenting with recurrent abdominal pain, hyponatremia, and psychiatric manifestations [1]. Prompt and accurate recognition is imperative, as untreated episodes may culminate in severe complications, including neurological deficits and life-threatening crises.

This case report has also been presented as a poster at the Society of Acute Medicine Spring Conference (SAM Belfast) held in Belfast on 02.05.2024 and 03.05.2024.

## Case presentation

**Figure 1:**
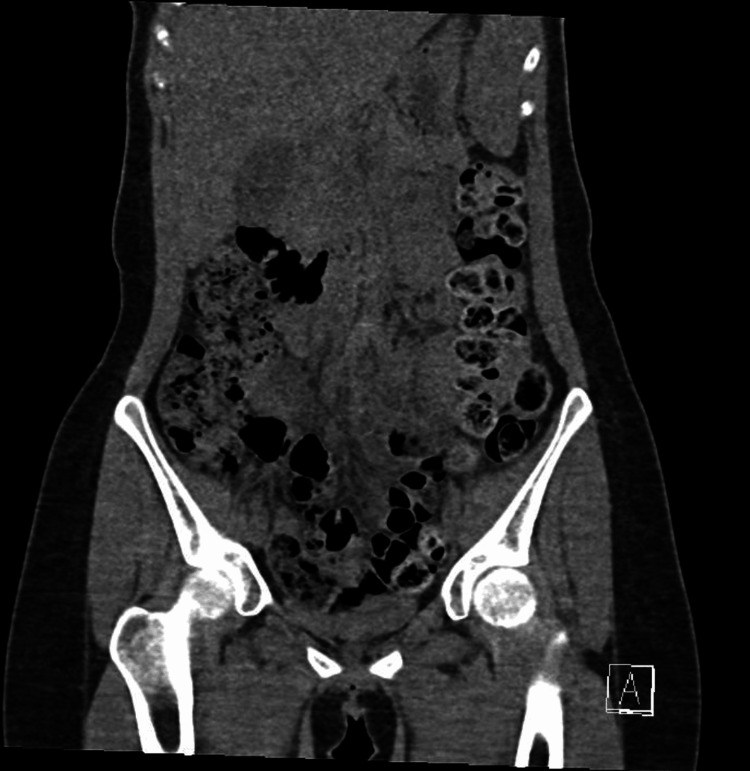
Apical view of CT imaging showing no acute abnormalities

**Figure 2:**
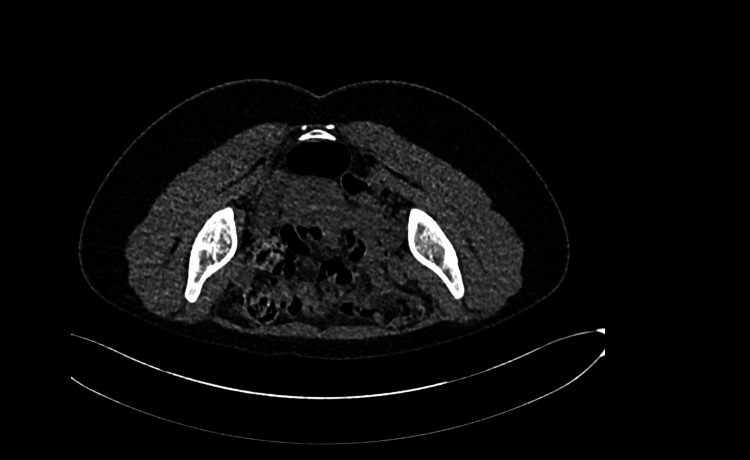
Unenhanced CT image showing no acute abnormalities

**Figure 3:**
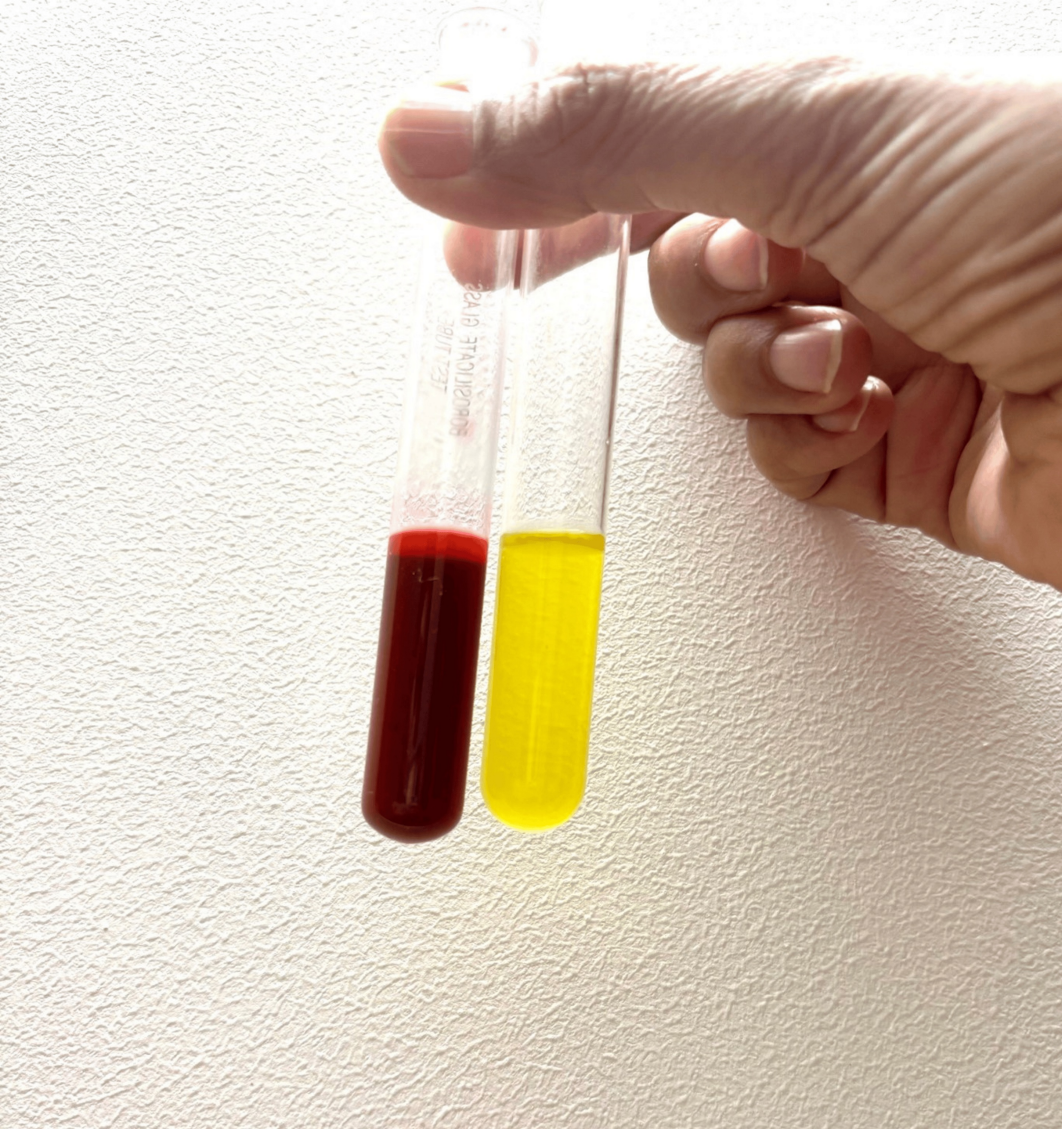
The test tube on the left indicates the urine sample collected from the patient showing typical reddish-brown colored urine after three hours of sunlight exposure during an acute attack of porphyria and the urine sample on the right shows the urine of a healthy adult female

A 20-year-old female presented to the Emergency Department with a clinical tableau characterized by severe abdominal pain, persistent vomiting, tachycardia, hypertension, and notable dark-colored urine. The patient appeared clinically dehydrated as well. A urine dip sample showed +++leukocytes and +++nitrates. Her past medical history included recurrent urinary tract infections (UTI) and a prior Intensive Care Unit admission for the syndrome of inappropriate antidiuretic hormone secretion (SIADH). She also had a history of anxiety, depression, and autism spectrum disorder. She was on treatment for a suspected UTI with Trimethoprim. Initial diagnostic evaluations, including unenhanced CT imaging (Figure 1 and Figure 2) and routine laboratory tests (Table 1), yielded no acute abnormalities; however, subsequent analysis of urine revealed elevated levels of porphobilinogen (PBG) and urine porphyrin/creatinine ratio (Table 2), thereby facilitating the diagnosis of acute intermittent porphyria (AIP) (Figure 3).

**Table 1:** Results of routine blood investigations WCC: white cell count; AKI: acute kidney injury; ALT: alanine transaminase; GFR: glomerular filtration rate

FULL BLOOD COUNT		RESULTS	UNITS	RANGE
	Hb	112	g/L	115-165
	WCC	3.3	10^9^/L	4.0-11.0
	Red Cell Count	4.63	10^12^/L	3.80-5.80
	Platelets	160	10^9^/L	150-400
	Neutrophils	1.7	10^9^/L	1.8-7.5
UREA & ELECTROLYTES				
	Sodium	139	mmol/L	133-146
	Potassium	3.9	mmol/L	3.5-5.3
	Urea	16.6	mmol/L	2.5-7.8
	Creatinine	77	umol/L	49-90
	AKI Stage	0		<0
BICARBONATE				
	Bicarbonate	24	mmol/L	22-29
CALCIUM & ALBUMIN				
	Total Calcium	2.67	mmol/L	2.10-2.60
	Albumin	44	g/L	35-50
	Adj. Calcium	2.65	mmol/L	2.10-2.60
PHOSPHATE				
	Phosphate	1.20	mmol/L	0.80-1.50
TOTAL PROTEIN				
	Total Protein	71	g/L	60-80
MAGNESIUM				
	Magnesium	0.84	mmol/L	0.70-1.00
CREATINE KINASE				
	Creatine Kinase	51	U/L	25-200
LIVER FUNCTION TEST				
	T. Bilirubin	13	umol/L	<21
	Alk. Phos	56	U/L	30-130
	ALT	22	U/L	<40
C-REACTIVE PROTEIN				
	C-Reactive Protein	<2	mg/L	<5
Estimated GFR				
	Estimated GFR	>90	mL/min/1.73m2	90-120

**Table 2:** Specific investigations supporting the diagnosis of AIP FBC: full blood count; U&E: urea and electrolytes; CRP: C-reactive protein; PBG: porphobilinogen; ALA: aminolevulinic acid

TEST	RESULT	REFERENCE RANGE
FBC, U&E	Within normal range	
CRP	<2	
Urine porphyrin/creatinine ratio	213 nmol/mmol Cr	<35nmol/mmol Cr
Urine PBG/creatinine ratio	37.4 umol/mmol Cr	<1.5 umol/mmol Cr
Urine ALA/creatinine	131 mmol/mol Cr	<3.3 mmol/mol Cr

The therapeutic approach encompassed aggressive hydration, analgesia utilizing morphine, codeine, and paracetamol, along with the cessation of trimethoprim, identified as a potential precipitant of her condition. Following a referral to the National Acute Porphyria Service (NAPS), she received intravenous Haem arginate for four days, resulting in marked symptomatic amelioration and subsequent discharge. Nevertheless, the patient experienced a recurrence of symptoms presenting to the Emergency Department four days after her prior discharge necessitating further admission for monitoring and pain management, highlighting the critical importance of preventing early discharge. This clinical vignette accentuates the necessity for heightened awareness and education among healthcare professionals regarding the atypical presentations of this rare and potentially life-threatening disorder, particularly in young females.

## Discussion

AIP is an autosomal dominant disorder caused by a deficiency of the enzyme porphobilinogen deaminase (PBGD). This deficiency leads to the accumulation of toxic metabolites, including porphobilinogen (PBG) and delta-aminolevulinic acid (ALA) [2]. This results in severe abdominal pain, vomiting, constipation, palpitations, urinary retention, and neurological manifestations like confusion, hallucinations, seizures, and peripheral neuropathy, which could lead to profound muscle weakness and, in some cases, psychiatric disturbances like insomnia, anxiety, and depression. AIP has an estimated incidence of 0.16 per million per year in the UK, making it a rare but crucial condition to recognize in acute medical care [3].

This case also delineates the rare concomitance of SIADH and AIP in a single patient, elucidating the complexity of their clinical manifestations, as very few cases have been verified in the English literature for the same [4-5]. In this case, a comprehensive diagnostic evaluation was undertaken, which included analyzing biochemical assays, including those from her previous medical records to affirm the presence of SIADH characterized by hyponatremia. Although the patient during this admission had normal sodium levels, she had a history of SIADH requiring ICU admission six months before her presentation. The patient’s clinical trajectory post-intervention underscores the significance of recognizing this dual pathology, ultimately leading to favorable outcomes. This report contributes a verified instance to the scant literature documenting the interplay between SIADH and AIP, thereby enriching the existing body of knowledge on this association. Furthermore, the identification of this rare co-diagnosis serves as a pivotal reminder for clinicians to consider the potential interplay of these conditions in differential diagnoses, advocating for a more nuanced understanding that could enhance diagnostic accuracy and therapeutic strategies.

Notably, the use of trimethoprim, an antibiotic contraindicated in AIP due to its potential to disrupt heme biosynthesis, was identified as a trigger for her acute crisis. It is vital for clinicians to be aware of medications that may exacerbate AIP to prevent future attacks [6].

Treatment and complications

Diagnosis can be challenging due to the nonspecific nature of symptoms, which may resemble other medical conditions. This ambiguity can lead to delays in treatment, increasing the risk of serious complications, including respiratory and cardiac failure [7]. While respiratory paralysis occurs in less than 10% of AIP cases, it can be life-threatening and requires immediate attention [3]. Timely recognition and management of such complications are essential. Treatment may involve mechanical ventilation and addressing the underlying porphyric crisis, with most patients recovering within days, though some may require extended respiratory support [8]. The primary treatment for AIP involves administering Haem arginate, which helps restore heme levels and reduce the accumulation of toxic metabolites [9]. Supportive care is also critical, including hydration using 0.9% sodium chloride, analgesia, and avoiding known precipitating factors such as smoking, consumption of alcohol, starvation, and the use of drugs that can induce attacks. Carbohydrate administration and avoiding fasting by administering regular meals is also vital in management. Liver transplantation is considered for patients facing frequent, severe acute porphyria attacks that are unresponsive to standard treatments. In conditions like acute intermittent porphyria (AIP), liver transplantation becomes crucial when these attacks result in a significant reduction in quality of life, reliance on ventilatory support, or challenges with venous access due to repeated hemin infusions [10].

## Conclusions

AIP presents a significant diagnostic challenge in acute medicine, requiring heightened clinical suspicion and early specialist intervention. Early diagnosis, including treatment with Haem arginate and avoiding precipitating factors, along with comprehensive supportive measures, are crucial in managing acute episodes effectively. Educational initiatives within Acute Medicine Departments can further enhance awareness and facilitate timely diagnosis, ultimately improving patient outcomes.

## References

[1] Stein P, Badminton M, Barth J, Rees D, Stewart MF (2013). Best practice guidelines on clinical management of acute attacks of porphyria and their complications. Ann Clin Biochem.

[2] Puy H, Gouya L, Deybach JC (2010). Porphyrias. Lancet.

[3] Findley H, Philips A, Cole D, Nair A (2012). Porphyrias: implications for anaesthesia, critical care, and pain medicine. Continuing Education in Anaesthesia Critical Care & Pain.

[4] Singh PS, Rawat R, Zafar KS (2014). Acute intermittent porphyria with syndrome of inappropriate antidiuretic hormone secretion (SIADH) and neurological crisis, successfully treated with haemodialysis. Int J Res Med Sci.

[5] Farese RV, Karsh SJ, Bidot-López P (1979). Acute intermittent porphyria associated with inappropriate antidiuretic hormone secretion, hypokalemic alkalosis, and secondary hyperaldosteronism. South Med J.

[6] Spiritos Z, Salvador S, Mosquera D, Wilder J (2019). Acute intermittent porphyria: current perspectives and case presentation. Ther Clin Risk Manag.

[7] Ramzan A, Cao JJ, Frazer JS, Stein P, Ahmad S (2023). A case of acute intermittent porphyria leading to severe disability in a young 21-year-old female. Cureus.

[8] Menegueti MG, Gil Cezar AT, Casarini KA, Muniz Cordeiro KS, Basile-Filho A, Martins-Filho OA, Auxiliadora-Martins M (2011). Acute intermittent porphyria associated with respiratory failure: a multidisciplinary approach. Crit Care Res Pract.

[9] Zhao L, Wang X, Zhang X, Liu X, Ma N, Zhang Y, Zhang S (2020). Therapeutic strategies for acute intermittent porphyria. Intractable Rare Dis Res.

[10] Seth AK, Badminton MN, Mirza D, Russell S, Elias E (2007). Liver transplantation for porphyria: who, when, and how?. Liver Transpl.

